# Draft genome sequence of *Polaribacter* sp. strain OB-PA-B3: a novel algal polysaccharide degrader isolated from the particle-associated fraction of Osaka Bay, Japan

**DOI:** 10.1128/mra.01287-25

**Published:** 2026-01-30

**Authors:** Chen-Yu Lin, Kenta Mitsunami, Kyota Miki, Yuto Hazui, Jota Suzuki, Ryoma Kamikawa, Takashi Yoshida

**Affiliations:** 1 Laboratory of Marine Microbiology, Graduate School of Agriculture, Kyoto University 12918, Kyoto, Japan; University of Wisconsin-Madison, Madison, Wisconsin, USA

**Keywords:** algal polysaccharide degradation, marine particle-attached bacteria, *Polaribacter*, CAZymes, flavobacteriaceae, particulate organic matter, Osaka Bay

## Abstract

A novel, algal polysaccharide-degrading *Polaribacter* strain, OB-PA-B3, was isolated from seawater in Osaka Bay, Japan. The genome sequence showed the highest average nucleotide identity (ANI) (91.9%) to *Polaribacter* sp. BM10 among available genomes. The 3.2 Mbp draft genome (29.8% guanine-cytosine [GC]) encodes carbohydrate-active enzymes (CAZymes), including a laminarin-utilizing enzyme.

## ANNOUNCEMENT

Members of the genus *Polaribacter* (family *Flavobacteriaceae*) are widely recognized as major degraders of complex polysaccharides derived from marine phytoplankton and particulate organic matter (1). In this study, we isolated a strain of *Polaribacter* sp. from the algal-attached fraction of seawater that is capable of utilizing algal polysaccharides.

Seawater was collected from Osaka Bay (34°19′28″ N, 135°7′15″ E), Japan at a depth of 5 m on 24 June 2025 and filtered through a 3.0-µm polycarbonate filter. The filter was immersed in 40 mL of sterile artificial seawater (Daigo SP; Wako, Japan) and incubated at 20°C overnight (2). After incubation, 1 mL of the suspension was serially diluted with sterile artificial seawater. Aliquots (100 µL) of the serially diluted suspension were spread onto Marine Agar 2216 (Difco) containing kanamycin (50 µg mL⁻¹) and a modified HaHa medium with 2 g L⁻¹ laminarin as the main carbon source (2, 3). Colonies were incubated at 20°C for 7 days under aerobic conditions. A single yellow-pigmented colony of OB-PA-B3 was purified by streaking three times on the same medium. Laminarin utilization by strain OB-PA-B3 was confirmed. The partial 16S rRNA gene sequence for OB-PA-B3 was PCR-amplified using the universal primers 515F-Y (5′-GTGYCAGCMGCCGCGGTAA-3′) and 926R (5′-CCGYCAATTYMTTTRAGTTT-3′) (4). The PCR products were subjected to Sanger sequencing (Eurofins Genomics, Tokyo, Japan). A BLASTn search revealed that the sequence of the isolated strain showed the highest sequence identity (99.73%) to that of *Polaribacter* sp. BM10 (GenBank accession number GCF_002005425.1) (5).

Genomic DNA of strain OB-PA-B3 was extracted using the DNeasy PowerWater Kit (Qiagen, Germany). Libraries were prepared using the MGIEasy Fast FS Library Prep Set V2.0 and DNB Rapid Make Reagent Kit with App-D primers (MGI Tech Co., Ltd.). Library quality was verified using Agilent 2100 Bioanalyzer (Agilent Technologies). DNBSEQ-T7 sequencer (MGI Tech Co., Ltd.) was used for 150-bp paired-end sequencing (Bioengineering Lab. Co., Ltd., Kanagawa, Japan), which yielded 4,156,353 paired-end reads.

Adapter trimming and quality filtering were performed using fastp v0.23.4 (6). The filtered reads were assembled with SPAdes v3.15.4 (7), and genome annotation was performed using the DFAST server v1.2.18 (8). The draft genome of strain OB-PA-B3 comprised 43 scaffolds, with an average genome coverage of 363×, an *N*_50_ value of 750,005 bp, a total length of 3,200,257 bp, a GC content of 29.8%, and 2,798 coding sequences. The average nucleotide identity (ANI) between OB-PA-B3 and *Polaribacter* sp. BM10 calculated using FastANI v1.33 (9) was 91.9%, which is below the commonly used 95% species boundary (9), indicating that OB-PA-B3 represents a novel species within the genus *Polaribacter*.

This new lineage utilizes laminarin, a key algal polysaccharide, demonstrating functional significance. The genome harbors numerous CAZyme families (10), including the laminarin-degrading enzyme GH17 (Glycoside Hydrolase Family 17) identified using HMMER v3.4 against dbCAN-HMMdb V14 (11). While its CAZymes profile was comparable to other *Polaribacter* genomes (12), OB-PA-B3 was isolated from a marine particle-attached fraction, suggesting adaptation to polysaccharide-rich microenvironments. These data provide a genomic resource for *Polaribacter* lineages from such environments.

## Data Availability

The genome sequence of *Polaribacter* sp. strain OB-PA-B3 has been deposited in the DNA Data Bank of Japan (DDBJ) under accession number BAAIIX010000000. The sequencing reads have been deposited in the DDBJ Sequence Read Archive (DRA) under accession number DRR751349. The DDBJ accession number for the 16S rRNA gene sequence of *Polaribacter* sp. strain OB-PA-B3 is LC899348.
